# P-2032. The Time to Return to Work in Healthcare Workers With COVID-19 Infection Treated With Ensitrelvir, a Novel Oral Inhibitor of 3C-Like Protease of SARS-COV-2

**DOI:** 10.1093/ofid/ofae631.2188

**Published:** 2025-01-29

**Authors:** Makoto Katsuta, Masatoshi Kitazono, Naohito Nagai, Hiroto Karibe, Yusaku Takahashi, Yasuko Ariwa, Takuhiro Sonoyama, Tomoyoshi Yamaguchi

**Affiliations:** Tokyo Rinkai Hospital, Tokyo, Tokyo, Japan; Tokyo Rinkai Hospital, Tokyo, Tokyo, Japan; Tokyo Rinkai Hospital, Tokyo, Tokyo, Japan; Tokyo Rinkai Hospital, Tokyo, Tokyo, Japan; Shionogi & Co., Ltd, Osaka, Osaka, Japan; Shionogi & Co., Ltd., Osaka, Osaka, Japan; Shionogi & Co., Ltd., Osaka, Osaka, Japan; Tokyo Rinkai Hospital, Tokyo, Tokyo, Japan

## Abstract

**Background:**

Early diagnosis and treatment with antivirals are important in COVID-19 management. This study aimed to investigate the time to return to work and COVID-19 symptom improvement in healthcare workers treated with ensitrelvir.

**Methods:**

A retrospective single-center, observational study was conducted to assess the effect of ensitrelvir on the time to return to work and the resolution of symptoms at the time of return in healthcare workers diagnosed with COVID-19 between June and September 2023 during the Omicron dominant period. The differences in patient background between the ensitrelvir treated group and no antiviral treatment group was not adjusted, and we conducted descriptive analysis. The criteria for returning to work were defined as improvement of symptoms and an antigen quantification test result of ≤ 100 pg/mL after the 6th day of onset.

**Results:**

A total of 102 healthcare workers were registered, and ensitrelvir was administered in 60 cases. The average number of days from onset of symptoms to ensitrelvir administration was 0.8 days. The female proportion was 76.7% and 78.6% in the ensitrelvir-treated and non-treated groups, respectively, with average ages of 38.2 and 30.6 years in each group, respectively. The severity of COVID-19 was mild in all cases. No side effects were observed in the ensitrelvir-treated group. The average time to meet the criteria for returning to work was 6.9 days in the ensitrelvir treated group and 7.7 days in the non-treated group. 35 patients (58.3%) in the ensitrelvir-treated group and 15 patients (35.7%) in the non-treated group experienced recovery (fever resolution and disappearance of COVID-19 symptoms) on the day of returning to work. Additionally, 25 patients (41.7%) in the ensitrelvir-treated group and 27 patients (64.3%) in the non-treated group experienced improvement (fever resolved but some symptoms remained) on the day of returning to work.

**Conclusion:**

This study showed that early treatment of COVID-19 with ensitrelvir may improve symptoms and reduce the number of days to return to work. The results suggest that early antiviral drug administration to healthcare workers may contribute to the maintenance and continuity of routine healthcare, thereby avoiding Healthcare workers shortage.

**Disclosures:**

Makoto Katsuta, Shionogi & Co., Ltd.: Lecture fees Takuhiro Sonoyama, MD, Shionogi & Co., Ltd.: Stocks/Bonds (Private Company) Tomoyoshi Yamaguchi, MD, Shionogi & Co., Ltd.: Lecture fees

